# Pharmacokinetics and Biological Activity of Cucurbitacins

**DOI:** 10.3390/ph15111325

**Published:** 2022-10-26

**Authors:** Eugenia Elisa Delgado-Tiburcio, Jorge Cadena-Iñiguez, Edelmiro Santiago-Osorio, Lucero del Mar Ruiz-Posadas, Israel Castillo-Juárez, Itzen Aguiñiga-Sánchez, Marcos Soto-Hernández

**Affiliations:** 1 Botany Department, Postgraduate College, Campus Montecillo, km 36.5 Carretera México-Texcoco, Texcoco 56230, Mexico; 2 Innovation in Natural Resource Management, Postgraduate College, Campus San Luis Potosí, Salinas de Hidalgo, San Luis Potosí 78622, Mexico; 3 Hematopoiesis and Leukemia Laboratory, Research Unit on Cell Differentiation and Cancer, FES Zaragoza, National Autonomous University of Mexico, Mexico City 09230, Mexico; 4 Department of Biomedical Sciences, School of Medicine, FES Zaragoza, National Autonomous University of Mexico, Mexico City 09230, Mexico

**Keywords:** cucurbitacins, absorption, distribution, metabolism, excretion, cancer

## Abstract

Cucurbitacins are a class of secondary metabolites initially isolated from the Cucurbitaceae family. They are important for their analgesic, anti-inflammatory, antimicrobial, antiviral, and anticancer biological actions. This review addresses pharmacokinetic parameters recently reported, including absorption, metabolism, distribution, and elimination phases of cucurbitacins. It includes recent studies of the molecular mechanisms of the biological activity of the most studied cucurbitacins and some derivatives, especially their anticancer capacity, to propose the integration of the pharmacokinetic profiles of cucurbitacins and the possibilities of their use. The main botanical genera and species of American origin that have been studied, and others whose chemo taxonomy makes them essential sources for the extraction of these metabolites, are summarized.

## 1. Introduction

Cucurbitacins are tetracyclic triterpenes produced by members of the Cucurbitaceae family and are divided into twelve categories, named from A to T [1]. However, research has focused mainly on cucurbitacins E (CuE), B (CuB), D (CuD), and I (CuI), which present differences between their acetylation patterns (C25) and OH groups (C2), although there are also various derivatives [2,3].

**Figure 1:**
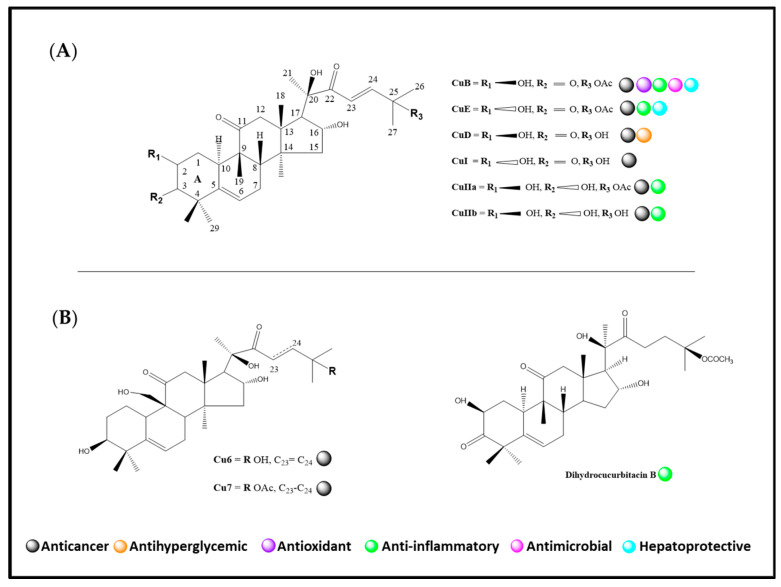
Overview of the chemical structure and biological properties of cucurbitacins. (**A**) The basic structure of this class of molecules is the core of cucurbitan (19-(10→β)-abeo-10α-lanost-5-ene), enumerated in the molecule’s center. Some of the main cucurbitacins in natural products are CuB, CuE, CuD, CuI, CuIIa, and CuIIb. The main differences are in the substitution pattern of ring A (C1–C5) and the side chain, where there may be unsaturation (C23–C25) and acetyl groups (C25). Additionally, alpha and beta hydroxyls are present at C2, C3, C7, C9, C16, and C20–C25. At the same time, unsaturation occurs at C23–C25, and the presence of keto groups in C2, C3, C11, C15, and C22. (**B**) Some derivatives important for their biological effects are shown.

All cucurbitacins have some biological activities (Figure 1), such as anti-inflammatory [4,5], hepatoprotective [6], antimicrobial [7], antioxidant [8], anthelmintic [9], antiviral [10], antihyperglycemic [11], and cardioprotective properties [12]. However, the main activity is its anticancer activity, the mechanisms of action of which include the induction of autophagy, pro-apoptosis, and control of cell proliferation [13,14].

However, cucurbitacins can present synergistic effects when combined, as is the case of CuB and CuE, which in their glycosylated form block the G2/M phase of breast cancer cells, inducing changes in cell morphology related to the alteration of actin filaments [15].

Cucurbitacin-producing plant species or their derivatives have great potential for developing alternative therapies in which complex mixtures or organic extracts are used [16,17]. However, it is necessary to know the dosage intervals and the pharmacological parameters to achieve their application [18,19]. Thus, this review describes and analyzes the pharmacological profiles of cucurbitacins available to date. Additionally, its biological activity and presence in other Neotropical plant species with bioactive potential are described.

The methodology was based on searching electronic databases (PubMed, Web of Science, Scopus, ScienceDirect, and Google Scholar). The keywords used were cucurbitacins, pharmacokinetics of cucurbitacins, anticancer and antiviral cucurbitacins, molecular mechanism of cucurbitacins, JACK/STAT3, biotransformation of cucurbitacins, distribution parameters of cucurbitacins, absorption of cucurbitacins in plasma, and cucurbitacins in Neotropical and Mesoamerican plants. The last access was in June 2022.

## 2. Pharmacokinetic Properties of Cucurbitacins

**Figure 2:**
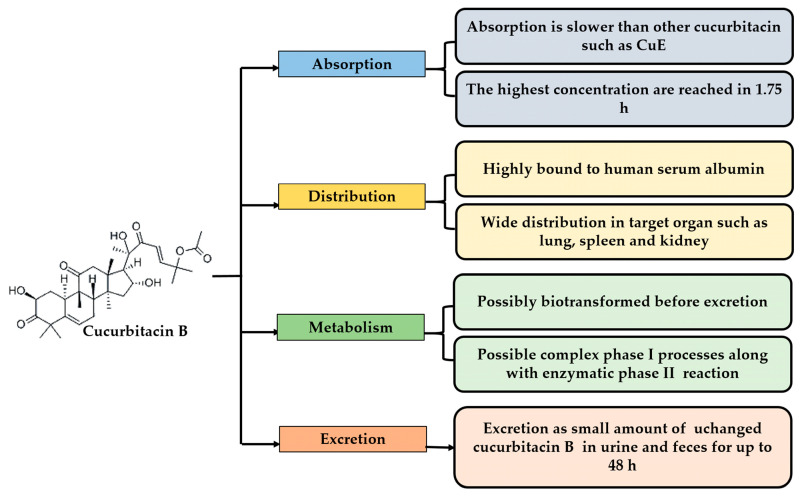
The pharmacokinetic properties of cucurbitacin B.

In recent years, the pharmacokinetics of some cucurbitacins have been reported; one of the most complete profiles is CuB and cucurbitacin IIa (CuIIa) [20,21,22] and, to a lesser extent, CuD, CuE, CuI, [23] and cucurbitacin IIb (CuIIb) [24]. It should be noted that most have been studied in a rat model, and some studies have been conducted in rhesus monkeys [25]. Although there has been gradual progress in research on the different types of cucurbitacins, it is still necessary to clarify some mechanisms of the pharmacokinetic profiles [20,26]. The most notable advances have been made in CuB (Figure 2), and differences in absorption, distribution, metabolism, and excretion have been observed among cucurbitacins despite their structural similarities [21].

### 2.1. Absorption

The different cucurbitacins show variable behavior patterns in their absorption pharmacokinetics, which could be determined according to the mode of administration, dosage, source of origin, and study models (Table 1). For example, the comparison between CuB from natural extracts (*Trichosanthes cucumerina*) or commercial sources in the same animal has revealed notable differences in the bioavailability range of 10% [21] and 1.37% [27], respectively. The C_max_ is 0.0097 and 0.03124 mg/L in 1 h, with 2 and 4 mg/kg extracted from natural sources, while C_max_ is 3.41 × 10^−5^ mg/L in 3 h with 8 mg/kg from commercial sources [21,28]. The published studies revealed that the bioavailability is low at only 10%, which can be associated with the compound’s solubility, digestion, gastrointestinal absorption, and membrane permeability [28].

It has been shown that the absorption of CuB is rapid, but the total absorption is reduced, an event that may be because hepatic or intestinal enzymes metabolize it before entering the systemic circulation. However, the concentration in plasma is directly proportional to the dose, which indicates that the pharmacokinetics of CuB are linear. Furthermore, CuE presents a higher absorption rate than CuB [26].

Absorption values may change in the presence of disease. Oral administration of the organic extract of *Hemsleya amabilis* in mice with gastric ulcers significantly increased the parameters of Cmax, Tmax, AUC, MRT, and T1/2, as well as the concentration of cucurbitacins in plasma. This effect may be due to increased gastric emptying caused by ulcerative lesions in animals [24].

### 2.2. Distribution

After absorption, some of the different cucurbitacins bind to human blood serum albumin (HSA) for further organ targeting. A study on cucurbitacin–albumin interactions with fluorescence spectroscopy and circular dichroism showed that CuB, E, and D have a strong affinity for albumin binding without a significant structural change in the HSA protein [29]. In addition, the thermodynamic criteria determined that for CuB, the forces are of hydrophobic and electrostatic type, while CuE mainly manifests hydrophobic forces, and in CuD (which has an OH group), it can form hydrogen bonds.

CuB had a high volume of distribution reflected in the wide dispersion toward target organs. Hunsakunachai et al., 2019 [21], showed a potential volume of distribution (V_d_ = 51.65 ± 39.16 L/kg) of CuB intravenous injection of 0.1 mg/kg, much higher than that reported for CuE (V_d_ = 27.22 L) at a dose of 1 mg/kg [23]. The high perfusion of the organs could explain the above behavior in combination with the high lipophilicity of CuB (X*log* P  =  2.6). Interestingly, organ distribution was in descending order to the lung, spleen, kidney, liver, small intestine, and, finally, to the stomach. It was reported that significant concentrations could remain for a long time in different organs. In the lung and spleen, a concentration up to 60 times higher than that in plasma was reported one hour after intravenous administration and 240 times higher after two hours [21]. CuIIa has been characterized by rapid distribution by intravenous administration [28].

Some investigations explore alternatives to improve the reorientation toward a specific organ. For example, its hepatoprotective effect is described among the multiple potential biological properties of CuB [30]. CuB-loaded galactosylated solid lipid nanoparticles were used to target the liver; as a consequence, CuB incorporated into the nanoparticles reached a liver concentration of 63.6% (2.5 times more than CuB alone), significantly improving liver capacity and increasing hepatoprotective potency [31].

### 2.3. Metabolism and Excretion

Some of the typical metabolic reactions of biotransformation of cucurbitacins involve phase I reactions, such as hydrolysis, oxidation, dehydration, and demethylation, and phase II reactions, such as glucuronide binding, as is the case for CuIIa [30], which shows a rapid and high elimination rate in the kidney in rats and rhesus monkeys [25,28]. However, some variables can modify the removal speed; for example, the clearance rate of CuIIa in diseased mice was much slower than that in healthy mice, possibly due to the CYP450 enzyme system, which prolonged storage time and decreased clearance time [24].

In the case of CuB, Hunsakunachai et al., 2019 [21], found less than 1% of this unchanged compound in feces after oral administration in rats in 48 h, indicating a possible metabolism before elimination. Although the biotransformation process is not yet well established, it is speculated that CuB could be involved in phase I processes in combination with phase II enzymatic reactions [21].

In contrast to the approaches mentioned above, Abbas et al., 2013 [32], studied the biotransformation of CuI, CuD, and CuE (phases I and II) by enzymes from human liver microsomes and UDP-glucuronosyltransferase isoforms. They reported the hydroxylation of cucurbitacins when incubated with liver enzymes. However, it was classified as low due to the presence of tertiary OH groups that slow down the rate of esterification reactions.

However, these studies revealed the production of cucurbitacin I by hydrolysis of cucurbitacin E. Additionally, the glucuronidation reaction detected UGT1A1 as the isoform with the greatest participation in the metabolism of the three cucurbitacins, followed by UGT1A7, 1A10, 1A6, and 1A8, and the glucuronidation rate was higher due to the presence of CuE and CuI. Finally, the sulfation activities of cucurbitacins I, D, and E were identified by the presence of sulfotransferases from human liver cytosols [32].

Although the biological effects of various cucurbitacins have been well documented, and recent pharmacokinetic studies of these components are still in progress, it should be noted that there are still several limitations in knowledge, or more precisely, the mechanisms are not yet fully clarified [20,21,33].

## 3. Therapeutic Efficacy of Cucurbitacin

There have been a large number of reports on the role of cucurbitacins in their medicinal and toxic properties. Among its most recognized effects is its anticancer potential through inhibition processes of the JAK/STAT3 signaling pathway, whose abnormal activation can direct cascade events involved in the development of cancer [34]. JAK (Janus kinase) is a family of tyrosine kinases: JAK1, JAK2, JAK3, and TYK2 [35], which, once activated by the interaction of cytosine and its receptor, undergoes phosphorylation in the accompanying sites of transcription factors, signal transducers and activators of transcription (STAT), to which it phosphorylates and, thus, becomes a transcription factor that translocates to the nucleus and regulates the expression of genes associated with proliferation mechanisms, such as cyclins and c-myc, and suppresses proapoptotic genes, such as survival, Bcl-xL, and Bcl-2 [36,37]. It has been described that the different members of the kinase family (JAK, JAK1, JAK2, JAK3, TYK2) can be constitutively activated in many hematopoietic malignancies, as well as in numerous types of cancer [38], such as bladder, colon, and cervical cancers and medulloblastoma [39], as well as leukemias [40] and lymphomas [41].

**Figure 3:**
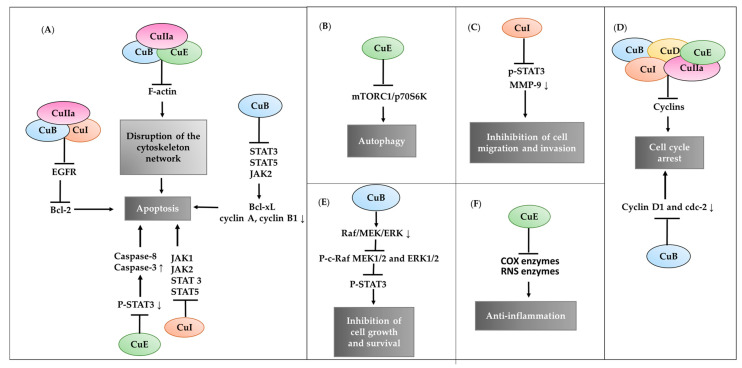
Most important biological action mechanisms of CuB, CuD, CuE, CuI, and CuIIa, where cucurbitacins have the following effects: (**A**) apoptotic, (**B**) autophagic, (**C**) cell migration and invasion, (**D**) cell cycle arrest, (**E**) inhibition of cell growth and survival, and (**F**) anti-inflammatory.

Evidence indicates that cucurbitacins are anticancer agents that prevent STAT3 DNA binding [42]. Since cucurbitacin treatments decrease the level of phosphorylated JAK or STAT3 and its downstream targets, such as Bcl-2, in cancer cells, the ability to stimulate apoptosis in cells has also been described [43] (Figure 3A). However, cucurbitacins may target different therapeutic targets to inhibit the growth of cancer cells.

In this context, it has been found that CuB inhibits the epidermal growth factor receptor (EGFR), prevents the growth of pancreatic cancer cells, and produces apoptosis through the negative regulation of anti-apoptotic proteins such as Bcl-2 and the increase in the amount of the proapoptotic protein Bim [44] (Figure 3A). CuE has been found to significantly affect apoptosis in bladder cancer through a decrease in the phospho-signal transducer and STAT3, which can trigger mitochondria-dependent pathways through sequential activation of caspase-8 and caspase-3 [45]. In addition, with CuB, arrest of the G2-M phase and induction of apoptosis in cancer cells was associated with the inhibition of JAK2, STAT3, and STAT5 by decreasing Bcl-xL [46] (Figure 3A).

The alteration of the actin cytoskeleton is another mechanism of cucurbitacins that promotes death in different types of carcinoma cell lines, possibly due to the direct alteration of the polymerization of the actin filaments [47] (Figure 3A). In addition, CuE activates autophagy in human cancer cells by downregulating mTORC1 signaling, an essential pathway in the regulation of autophagy, which is a promoter of the autophagy mechanism through activation of ULK1, causing mTORC1 inhibition [48] (Figure 3B).

In addition to anticancer processes, cucurbitacins play an important role in preventing the migration and invasion of cancer cells. CuI has been reported to mitigate invasion in colon cancer cell lines associated with the downregulation of STAT3 phosphorylation and MMP-9 expression, an enzyme associated with cell invasion [49] (Figure 3C).

Another essential feature of cucurbitacins is their ability to disrupt the cell cycle of cancer cells [45]. One of the main mechanisms of action is the inhibition of genes encoding cyclins. CuB has been reported to reduce cyclins D1 and cdc-2, which are key to developing the G2/M phase in human hepatocellular carcinoma cells (Figure 3D).

Cucurbitacins also inhibit the Raf/MEK/ERK signaling pathway in leukemic cells, which regulates cell proliferation, growth, differentiation, and senescence. Chan et al., 2010 [50], reported that CuB could interact with the Raf/MEK/ERK and JAK/STAT3 pathways in leukemia cells, thereby inhibiting their growth (Figure 3E).

The role of cucurbitacins in inflammatory properties has been reported. CuE inhibits the COX and RNS enzymes, which are related to the severe inflammatory response in various chronic disorders [4] (Figure 3F).

**Figure 4:**
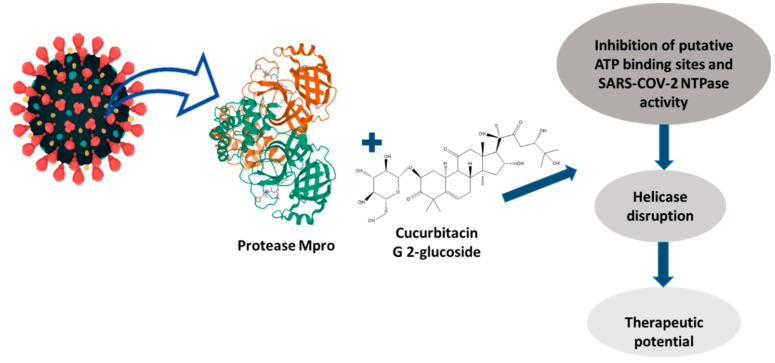
Possible mechanism of action of cucurbitacin G2-glucoside against protease Mpro SARS-CoV-2.

The antiviral potential of CuB, CuE, and CuD against hepatitis C virus (HCV), bovine viral diarrhea (BVDV), and hepatitis C virus (HCV) has been reported. However, as a consequence of the contingency problems caused during the last two years due to severe acute respiratory syndrome coronavirus 2 (SARS-CoV-2), the working group of Kapoor et al., 2020 [10], analyzed 16 cucurbitacin analogs and their interaction with the main protease protein (Mpro) of the coronavirus, demonstrating that all proteins bind efficiently and specifically with cucurbitacin G 2-glucoside, which suggests the possibility of inhibiting the activity of helicase enzymes involved in the virus replication cycle (Figure 4).

### 3.1. Biological Action of Cucurbitacin B

CuB, (2β,9β,10α,16α,23*E*)-25-(acetyloxy)-2,16,20-trihydroxy-9-methyl-19-norlanosta-5,23-diene-3,11,22-trione, is one of the most abundant classes of cucurbitacins and is one of the most explored cucurbitacins due to its role in biological systems [51]. There have been numerous reports on the anticancer properties of cucurbitacin B (Table 2), which include the inhibition of tumor cell lines [52,53], as well as its anti-inflammatory properties [8,54]; antimicrobial and antiviral properties [55]; antiaging properties [56]; antidiabetic properties [57]; antihypertrophic and antifibrosis properties [12]; and its actions as a memory protector of mice in APP/PS1 and neurogenesis inducer [58].

One of the most interesting attributes of CuB is its potential as an anticancer agent, both in vivo and in vitro, which includes growth inhibition, cell cycle arrest in the G2/M phase, and the induction of apoptosis in numerous cancer cell lines. It has been implied that these effects could derive from the disruption of STAT3 phosphorylation, Bcl-2, and cyclin B1 expression [59]. In addition, modifications in the composition of the cytoskeleton have been demonstrated in myeloid leukemia cells [60].

Regarding breast cancer, Liang et al., 2018 [61], showed that CuB inhibits the adhesion and viscoelasticity of various cell lines, reduces cell abnormalities, and prevents the invasion and migration of malignant cells associated with the expression of F actin/vimentin/FAK/vinculin (directs the distribution and arrangement of the cytoskeleton). Similarly, CuB inhibited the RAC1/CDC42/RhoA signaling pathway (key elements in cell viability and migration) and prevented the generation of metastasis in cancer cells.

CuB-related methylation studies could represent a new pathway through which to counteract breast cancer. Aberrant methylation is a trigger in the development of tumors, and for this reason, special attention has recently been paid to the role it plays in the development of cancer cells caused by the abnormal expression of genes due to the presence of epigenetic factors. In a study by Dittharot et al., 2019 [53], CuB extracted from *Trichosanthes cucumerina* acted as a hypermethylation agent and suppressed the expression of the oncogenic promoters of c-Myc and cyclin D1; therefore, it is considered a potential therapeutic agent against breast cancer.

It has been reported that CuB induces apoptosis and prevents proliferation in lung cancer cell lines through the disruption of the specific inactive transcript of lncRNA X (XIST) [62], which at irregular levels is associated with the presence of tumors (colon and breast cancer) [63,64], also confirming that CuB induces apoptosis through upregulation of miR-let-7c [62].

Additionally, CuB may benefit osteosarcoma, and one of the most diagnosed tumors in children and young adults [65,66] revealed that osteosarcoma cells (U-2 OS) treated with CuB downregulated the phosphorylated ERK1/2, p38, and JNK. Substantial decreases were also observed in the levels of p38 and ERK1/2 and levels of JNK and p-JNK, thus demonstrating that the decrease in the expression of the MAPK signaling pathway is an important mechanism in the stimulation of apoptosis of U-2 OS cells.

Another regulatory mechanism in which CuB may have relevant effects in oncogenesis has been described by Qin et al., 2018 [67]. CuB prevents proliferation and invasion in human glioblastoma multiforme (GBM) cell lines, in addition to downregulating the expression of the oncoprotein CIP2A and its downstream signaling molecule phospho-Akt, suggesting that CuB could be a potential inhibitor of CIP2A.

Regarding its anti-inflammatory effects, CuB reduced inflammatory responses in conditions such as periodontitis. As reported by Zhong et al., 2020 [5], based on an experimental periodontitis induction in rats through the ligation method, CuB treatments (12.5 mg, 25 mg, and 50 mg kg^−1^ body weight) for 12 days showed a significant decrease in alveolar bone loss through the regulation of RANK/OPG levels, as well as the reduction of inflammatory responses in periodontitis in a preclinical trial.

CuB activity may also have antiaging effects. Lin et al., 2019 [56], found that treatments with CuB yielded a significant increase in the replicative rate and the chronological life in survival years of the yeast mutant strain *Saccharomyces cerevisiae* K6001 via modulation of autophagy and antioxidant activity to increase the longevity of yeast.

**Figure 5:**
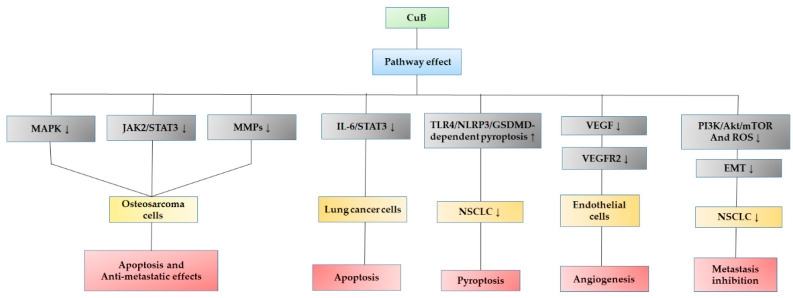
Recent anticancer effects described on different cell signaling pathways by CuB. Upregulation (↑) and downregulation (↓).

Finally, the anticancer CuB mechanism is not limited to JAK/STAT pathway disruption but also induces disruption of many other types of signaling that increase tumor cell growth, such as NFκB [68], PI3K/Akt/mTOR [44], MAPK/ERK [65], or Wnt/β-catenin progression [69]. It also induces the expression of those tumor suppressor genes, such as p53, and, thus, induces apoptosis, regulates cell survival, or inactivates the overexpression of oncogenic agents [70]. Some other important pathways involved in the anticancer effect in different cell lines have recently been described (Figure 5), such as the induction of apoptosis through the inhibition of matrix metalloproteinases (MMPs) and interleukin-6 (IL-6) [62,65]; pyroptosis death by inhibition of non-small cell lung cancer (NSCLC) by triggering TLR4/NLRP3/GSDMD-dependent pyroptosis [71]; anti-angiogenesis effects by triggering angiogenesis through the mitochondrial signaling pathway, which causes the inhibition of vascular endothelial growth factor (VEGF) and subsequent inactivation of vascular endothelial growth factor receptor 2 (VEGFR2) in endothelial cells [72]; and inhibition of metastasis through the inactivation of TGF-β1-induced epithelial–mesenchymal transition (EMT) in NSCLC through regulation of the ROS and PI3K/Akt/mTOR pathways [73]. However, although there are an increasing number of new conditions in which CuB can be a potential treatment, particularly in cancer, more clinical studies are still needed to define its effectiveness.

### 3.2. Biological Action of Cucurbitacin D

CuD has been described as a potential antitumor agent in several cancer models due to its ability to induce apoptosis through the inactivation of NF-κB and STAT3 or to produce autophagy in some tumor cell lines [89,90]. CuD is also known to control cancer cell proliferation, migration, and spread [91,92]. It has been observed to promote immunomodulatory activity in macrophages since it increases the production of IL-1β induced by lipopolysaccharides, which stimulates inflammasome activation [93].

Authors such as Sikander et al., 2019 [92], studied the efficiency of CuD against prostate cancer by reprogramming the metabolic switch and molecular interaction with the GLUT1 receptor. Among the main results, they observed that CuD has significant cytotoxic effects on prostate cancer cell lines (PrCa) since it stops the progression of the cell cycle in the G2/M phase. Another important finding is that a lack of glucose is a sufficient mechanism to generate growth arrest and cell death in PrCa. Similarly, CuD reduced the expression of GLUT1 since its overexpression is correlated with glucose uptake. This showed that CuD could reprogram glucose metabolism, leading to growth inhibition of metastatic PrCa cells.

Studies conducted by Ku et al., 2018 [94], revealed that CuD increased p-p53 levels but directed the downregulation of p-Akt, p-NF-κB, and p-Stat3 in breast cancer cell lines MCF7, SKBR3, and MDA-MB. The results of this assay were comparable to those of the assay for drug doxorubicin, which individually failed to lower p-Akt and p-Stat3 levels. The combination of doxorubicin and CuD increases the potential effect by increasing the levels of p-p53 and disrupting the expression of Akt, NF-κB, Stat3, and Bcl-2.

Zhang et al., 2018 [95], focused on the growth and death of three cell lines related to gastric cancer (AGS, SNU1, and Hs746T) under CuD treatment. When the antiproliferative potential was highlighted, it induced the production of ROS and, therefore, the induction of apoptosis. The presence of CuD increased the intracellular levels of Ca^2+^ and ATP. CuD activated the mitochondrial apoptosis pathway, and with its positive expression of Bax, CuD modulated the activation of the iNOS pathway, which produced ROS and nitric oxide molecules, to activate the apoptosis of cancer cell lines.

Recently, the role of CuD as an adjuvant therapy has been investigated. Kodidela et al., 2021 [96], studied the anti-HIV effect of CuD in HIV-infected macrophages using the blood–brain barrier (BBB) as an in vitro model. The results showed that CuD decreased viral load significantly, and there was also a decrease in the proinflammatory cytokine IL-1β and HIV replication; thus, CuD can be considered a potential compound to be used as adjuvant therapy with two purposes: to decrease brain toxicity from antiretroviral failure and to prevent HIV in the brain.

### 3.3. Biological Action of Cucurbitacin E

CuE is another of the most abundant forms of cucurbitacin, the biological activity of which is mainly associated with anticancer properties [97] (Table 3), but it has also been described as having anti-inflammatory [4], antiviral [98], and hepatoprotective attributes [99]. 

The anticancer mechanism of CuE is described by suppressing the activation of the transducer and transcriptional activator 3 (STAT3). It was also observed that p53 and p21 could be increased in cancer cells treated with CuE and produce alterations in the levels of a protein associated with the G2/M phase in cancer cells and, therefore, arrest in the cell cycle. Similar studies are highlighting the effect of this cucurbitacin on other signaling pathways. For example, He et al., 2017 [97], evaluated the effect of CuE targeting human colon cancer cells (LNCaP), demonstrating cytotoxic action, suppression of cell viability, and activation of apoptosis via an increase in cofilin-1, AMP-activated protein kinase, p53, and expression of the caspase-9 protein; therefore, it is proposed that the mechanism of action of CuE is exerted through the signaling of cofilin-1, mTOR, AMPK, p53, and caspase-9.

Other authors, such as Saeed et al., 2019 [100] developed multiple studies of CuE isolated from *Citrullus colocynthis* against drug-resistant tumor cell lines. Three members of the ABC transporter superfamily related to multidrug resistance, P-glycoprotein (P-gp), breast cancer resistance protein (BCRP), and ABCB5, were investigated. It was found that lines positive for overexpression of P-gp and BCRP show cross-resistance to CuE, while those that overexpress ABCB5 show chemosensitivity to this cucurbitacin, demonstrating that CuE is not beneficial for patients with tumors overexpressing P-gp and BCRP.

CuE has played an important role in chronic diseases such as asthma. Recently, Shang et al., 2019 [101], investigated the role of CuE in the inflammatory process of asthma due to its potential to inactivate the NF-κB pathway and, thus, counteract the production of proinflammatory cytokines such as TNF-α and INF-γ, for which the protective effect of CuE on the human bronchial epithelial cell line BEAS-2B was measured by means of an in vitro assay, in which the inflammatory response in epithelial cells exposed to lipopolysaccharides (LPS) simulated asthmatic conditions. The results showed that the administration of CuE inhibited the production of inflammatory cytokines induced by LPS, such as TNF-α, IL-6, and IL-8, by inhibiting the activation of HMGB1, TLR4, and p-p65 NF-κB, as well as reducing the release of mucin 5AC (MUC5AC); therefore, CuE could be considered a promising agent in asthma therapy.

### 3.4. Biological Action of Cucurbitacin I

It has been reported that in human breast carcinoma and lung adenocarcinoma cell lines, CuI inhibits STAT3 activation [106]. In a study conducted by van Kester et al., 2008 [107], it was shown that CuI downregulates the dose-dependent STAT3 phosphorylation and induction of apoptosis by CuI in Seax cells derived from the aggressive cutaneous lymphoma line of CD4+ T cells with tumor cells (Sz).

Dandawate et al., 2020 [52], described the effect that CuI promotes against different colorectal cancer (CRC) cell lines, thus demonstrating that CuI suppresses cell proliferation in CRC. This revealed G2/M cell cycle arrest within 24 h, coupled with a significant decrease in cyclin B1 and cyclin-dependent kinase 1 (CDK1). Among other affected compounds were the proteins involved in the transition of cells from the S phase (cyclin A2, CDK2, Wee1, and CDC25C to G2/M), which provided greater support for the interruption of the cell cycle in the lines, also highlighting important effects for triggering apoptosis. Another interesting finding was that CuI produced an increase in cleaved caspase 3 and PARP proteins, registering a higher expression of the proapoptotic marker Bax and, at the same time, a decrease in the antiapoptotic markers Bcl-xL, Mcl-1, and Bcl-2; therefore, this could suggest that the activation of apoptosis occurs through caspase in CRC cells. In addition, CuI showed binding to the ankyrin domain of the Notch receptor, so it is inferred that the suppressive effect of CuI in colon cancer is mediated by inhibiting the Notch signaling pathway.

In another study carried out by Li et al., 2019 [108], the effect of CuI on endoplasmic reticulum stress (ERS) was studied, where ERS is a fundamental response to confer protection against any alteration in the endoplasmic reticulum and, therefore, is an important factor in the development of tumors. Thus, CuI showed potent anticancer action by inducing apoptosis due to excessive induction of ERS and C/EBP homologous protein (CHOP) and caspase-12-dependent ERS-generated apoptosis. In addition, the ERS, IRE1α, and PERK pathways, as well as CHOP, were activated after CuI treatment in SKOV3 and PANC-1 cancer cell lines. Therefore, CuI could have potential for the development of new anticancer therapeutic approaches.

Ni et al., 2018 [109], reported that CuI decreased cell viability and colony formation through the activation of apoptosis in lung cancer lines (A549) and the production of autophagic vacuoles with increased apoptosis by inhibiting ERK activation as well as the downstream phosphorylation of mTOR and STAT3. Although much remains to be elucidated regarding the profile of CuI and several of its biological effects, to date, a possible therapeutic window for the development of anticancer phytopharmaceuticals is provided.

### 3.5. Biological Action of Cucurbitacin IIa

CuIIa, also named hemslecin A or 25-*O*-acetyl-23,24 dihydrocucurbitacin F, is the most important bioactive component in *Hemsleya* species and has in its structure five active sites: C-2 hydroxy, C-3 hydroxyl, C-16 hydroxyl, B-ring double bond, and C-22 aldehyde groups. All five of these are widely used for derivative synthesis [22,24,110]. CuIIa has multiple biological effects in pharmacological studies as an anticancer [111], anti-inflammatory [112], antiviral [113], or antidepressant agent [114].

Zhang et al. (2019) [115] evaluated the effect of CuIIa on the lung cancer cell line A549. They reported that CuIIa is a repressor of mitogen-activated protein kinase MAPK signaling through competitive inhibition of the EGF-binding site on the EGFR protein. As a result, transcription, agglomeration, and phosphorylation of signaling constituents (such as STAT3) are altered, inducing apoptosis and cell cycle arrest in the G2/M phase of cancer cells.

An assay conducted by Boykin et al. (2011) [111] described that CuIIa eliminates the distribution of cancer cells in vitro through the suppression of the actin cytoskeleton and the pathways involved in survivin and PARP, mediators of the apoptosis process in prostate cancer cells (CWR22Rv-1). In addition, the cell cycle was interrupted with the reduction of phospho-histone H3 and survivin, suggesting an important correlation between mitosis and survivin together with the p53 and p21 pathways to enhance the anticancer action of CuIIa. In addition, since the function of PARP is limited, there is less capacity to repair damaged DNA, resulting in a much faster process of apoptosis through the p53 and p21 pathways. This demonstrated that the CuIIa biological effect is not conferred in a conventional manner to other cucurbitacins by not suppressing JAK2/STAT3 phosphorylation; instead, the trigger is mediated by suppression of actin cytoskeleton arrest and related signaling pathways.

Studies conducted by Zhou et al., 2017 [114], showed that CuIIa could pass through the blood–brain barrier and had antidepressant-like effects in trials with mouse models, in which they were subjected to chronic unpredictable mild stress (CUMS) through elevated plus maze, open field, forced swimming, and tail suspension (induction of behavioral changes) tests for antidepressant treatments. In the tests described, CuIIa treatment restored the irregular behavior of the mice through more significant locomotor activity and less immobility time. Decreased levels of brain-derived neurotrophic factor (BDNF) are known to cause erratic synaptic plasticity, which can subsequently lead to depression, suggesting that CuIIa has a potential use in antidepressant diseases and as a neuroprotectant by downregulating the CaMKII-CREB-BDNF pathway.

The above evidence demonstrates that the diversity of biological effects of CuIIa may be implicit through the JAK2/STAT3 [116], ERBB MAPK [115], and CaMKII α/CREB/BDNF pathways [114], among others; however, there are still mechanisms to elucidate and explore its effects on other chronic diseases [22], which would contribute to its relevance as a phytopharmaceutical. 

### 3.6. Biological Action of Cucurbitacin IIb

CuIIb, named 23,24-dihydrocucurbitacin F or hemslecin B, is isolated from the plant *Hemsleya amabilis* [117]. It has been described for its effects on the induction of apoptosis and cell cycle suppression through regulation of the EGFR/MAPK pathway or by inhibition of STAT3 [118,119] and apoptotic activity in cancer cell lines of the cervix or lung [119] and anti-inflammatory activity [117].

CuIIb has a relevant effect on systemic lupus erythematosus (SLE) since it breaks the imbalance between Th17 and Treg cells and is involved in the pathogenesis of SLE. Wu et al., 2020 [120], evaluated CuIIb regulation in Th17/Treg cells using in vivo mouse models of SLE. After treatment with CuIIb, the production of Treg cells was increased in mice, but the opposite effect was produced in Th17 cells. In addition, CuIIb induced the expression of foxp3 but repressed RORγt in SLE mice and repressed IL-6 and IL17, which were highly expressed, and induced IL-10 TGF-β in lymphocytes, which was expressed at low levels in lymphocytes from SLE mice. Thus, CuIIb mitigated the kidney damage caused by SLE. 

Liang et al., 2021 [118], described the antiproliferative activity of CuIIb in A549 lung cancer cell lines through the STAT3 pathway that is modulated by the mitochondria and is caspase-dependent, in conjunction with an alteration of cellular activity in the G2/M phase, which is also attributed to the ability to intervene in the signaling of the mitogen-activated protein kinase/EGFR (MAPK) pathway. Finally, it was demonstrated by molecular docking that CuIIb-EGFR binding is due to hydrophobic and hydrogen bonding interactions, strongly supporting CuIIb as a potential EGFR TKI.

### 3.7. Cucurbitacin Derivatives and Their Biological Activity

The chemical structure of cucurbitacins can be modified to obtain various derivatives; therefore, 200 derivatives of these compounds have been described [1,22,121]. These derivatives include substitutions at C2 or C3, isomerization, deoxidization, or dihydro derivatives [122]. Remarkably, derivatives of cucurbitacins B, D, E, and I have mainly been studied for their potential anticancer effect [121].

Some important studies of derivatives, such as 23,24-dihydrocucurbitacin B, have been highlighted for their antiarthritic effects in mice that were treated with this compound, which produced an anti-inflammatory effect by downregulation of proinflammatory enzymes, such as elastase, cyclooxygenase-2, and nitric oxide synthase-2, and mediators, such as tumor necrosis factor-α and interleukin-1β, without modifying macrophages. In addition, dihydrocucurbitacin B was able to decrease cell inflammation, infiltration, joint damage, and osteoclast activity [123].

In another study conducted by Ren et al., 2012 [124], the antiproliferative effect of 23,24-dihydrocucurbitacin F, another derivative, on human prostate cancer cells was studied, with the results demonstrating that this compound had the ability to stop cell growth and inhibit the cell cycle in the G2/M phase, which could be directed through the induction of actin aggregation and cofilin-actin rod formation by disruption of cytokinesis with minimal effect on microtubules.

In addition, it has been possible to determine an important antihyperglycemic effect of 23,24-dihydrocucurbitacin D and 2-*O*-*β*-glucopyranosyl-23,24-dihydrocucurbitacin D isolated from *Ibervillea lindheimeri* in diabetic mice, where both compounds reduced blood glucose in diabetic mice compared with healthy controls, due to translocation of glucose transporter type 4 (Glut4) to the plasma membrane (PM) on epididymal adipose tissue (EAT) as its main target; however, these cucurbitacins also produce activation of AMP-induced protein kinase (AMPK) in soleus muscle (SM) or dual activation of AMPK and protein kinase B (AKT) in EAT independent of insulin. In addition, both cucurbitacins had the ability to bind to different sites of activation of cystathionine β-synthetase (CBS) and showed a high affinity to the binding site of the competitive inhibitor AKT G98, which possibly contributed to the activation in adipose tissue [11].

In a recent study by Qing et al., 2022 [17], it was found that postmodified derivatives of cucurbitacin C (CuC) from *Cucumis sativus,* which were named Cu6 and Cu7, showed growth inhibition capabilities against the tumor cell lines HepG2, A549, DU145, and HCT116 by apoptosis induction.

However, it has been found that derivatives of cucurbitacins can have a different effect from their predecessors; for example, it has been described that cucurbitacin D has a significant effect against different cancer lines of lung cancer or human colon cancer, unlike its derivative 2-*O*-glucoside of cucurbitacin D, which does not show any relevant anticancer activity in this way. It is inferred that the different types of derivatives of cucurbitacins will not necessarily have the same effect as that of the class to which they belong [1].

## 4. Screening and Methodologies for the Identification of Cucurbitacins

The optimization of the extraction method for the isolation and identification of bioactive compounds is essential in analytical studies [125]. Due to the biological potential of cucurbitacins, it has been necessary to develop reliable estimation techniques to determine the content of cucurbitacins in different plant species. Previous studies have described the isolation and characterization of cucurbitacins in different cucurbits, such as *Corallocarpus epigaeus* [126], *Blastania* species [127], *Cucumus callosus* [85], *Momordica charantia* [128], *Ecballium elaterium* [129], and *Sechium edule* [130]. 

Cucurbitacins can be extracted by usual methods, such as maceration and subsequent fractionation with solvent, open column chromatography, and thin-layer chromatography. The most important factors to consider in this process are the solvent’s intrinsic characteristics and the sample´s properties. The best option is to use solvents of higher polarity. Among the most appropriate for the extraction of cucurbitacin are methanol and ethanol; however, it has been decided to partition these solvents into less soluble molecules such as aglyconic cucurbitacins [125].

In addition, there are other less conventional methods, such as supercritical fluid extraction (SCFE), ultrasonic-assisted extraction (UAE), and microwave-assisted extraction (MAE), which may provide a different option in the extraction of cucurbitacins, where these main factors must be considered for efficient development with these techniques, namely, the sample matrix and the type and location of all the compounds in the matrix; however, the optimization of these cucurbitacin methods is still in the trial stage [131]. 

Attar et al., 2018 [125], chose acetone as the ideal solvent for obtaining a higher yield in mesocarp with Soxhlet extraction of CuI from *Lagenaria siceraria*. Just as it was found that conventional methods, such as Soxhlet extraction at 50 °C, which are highly efficient as well as fast and reliable, the use of chromatographic and spectrometric techniques, such as thin-layer chromatography (TLC), Fourier-transform infrared spectroscopy (FT-IR), and liquid chromatography–mass spectrometry (LC–MS), are very useful tools that enabled corroboration of the presence of CuI and other metabolites of interest. Using the same strategy, Attar et al. (2022) [127] described a successful extraction of CuI, CuB, and CuE isolated from *Blastania* species by RP-HPLC for their detection. In addition, a correlation was found between the content of terpenoids and their biological activities, such as antioxidant, antidiabetic, and anticancer activities, which were higher due to the higher content of these compounds.

## 5. Perspectives of Cucurbitacins from Plants of Mesoamerican Origin

Cucurbitacins are compounds described in numerous plant members, such as the Begoniaceae, Cruciferae, Datiscaceae, Desfontaiuniaceae, Euphorbiaceae, Elaeocarpacae, Scrophulariaceae, Polemoniaceae, Primulaceae, Rubiaceae, and Sterculiaceae families [132]; however, they are mainly found in the Cucurbitaceae family in the genera Bryonia, Cucumis, Cucurbita, Luffa, Echinocystis, Lagenaria, Citrullus, and Sechium, as well as in Momordica [130,132].

The Cucurbitaceae family has gained international relevance because, in this group, there are many domesticated species of great interest, mainly due to their economic importance in human and medicinal consumption. The family is composed of 965 plant species in 95 genera [133,134]. Countries such as Mexico stand out with wide biodiversity, as it is composed of up of 137 taxa (which includes species and infraspecific entities), unlike other Latin American countries, such as Guatemala and Panama, with a record of 53 species, Peru with 104, and Venezuela with 84. It should be noted that even Mexico is a center of domestication and diversification of pumpkins. The best represented native genera are *Cucurbita* and *Sicyos* (with 15 species and intraspecific entities each), *Cyclanthera* (14 species), *Apodanthera* (eight species), and *Echinopepon* and *Ibervillea* (with seven species each) [133]. 

The species *Cucurbita pepo* is the most investigated plant due to its content of cucurbitacins [134], and it should be noted that the content of cucurbitacins present may vary, depending on factors such as the specific structure of the plant and the stage of development, but they are generally found in the roots, stems, cotyledons, leaves, and fruits, although a higher content has been described in fruits at maturity and roots that increase over time [132,134].

Rehm et al., 1957 [135], studied the distribution of different types of cucurbitacin in different plant parts of different cucurbit species and found that each type of cucurbitacin such as CuB or CuE is mainly concentrated in the roots, but can also be found in combinations such as CuD and CuB or CuI and CuE. In the cotyledons, CuB or CuE is predominant, so, apparently, these types of cucurbitacins are the first bitter constituents formed when growth begins. 

In a study by Metcalf et al., 1982 [136], the concentrations of cucurbitacins in 18 plant species were studied, and they found high concentrations of CuB and CuD in the absence of CuI and CuE in bitter-tasting wild genotypes, such as *Cucurbita andreana*, *Cucurbita ecuadorensis*, *Cucurbita gracilior*, *Cucurbita *undelliana**, *Cucurbita palmeri*, *Cucurbita sororia,* and *Cucurbita pedatifolia*, unlike the species *Cucurbita cylindrata*, *Cucurbita foetidissima*, *Cucurbita martinezii*, and *Cucurbita okeechobeensis,* in which CuE and CuI are present, and a third group that included *Cucurbita ficifolia*, *Cucurbita maxima*, and *Cucurbita moschata,* which exhibited the presence of E-glycosides.

Other members of the Cucurbitaceae family that have become relevant due to the content of cucurbitacins are *Sechium edule*, an edible species whose center of origin is Mexico. Some varieties, such as Perla Negra, have exhibited type B, D, E, and I cucurbitacins, with D being the most predominant concentration. These components are attributed to part of their antineoplastic activity carried out in cancer cell lines such as HeLa. Additionally, Riviello-Flores et al., 2018 [130], revealed the presence of CuB, CuD, CuE, and CuI in *Sechium edule var. virens levis* and *Sechium edule var. nigrum spinosum* in ethanolic extract, highlighting the content of CuD. Recently, Cadena-Iñiguez et al., 2022 [137] again found the presence of CuB, CuD, CuE, and CuI in dichloromethane extract from the genotype *S. edule var. amarus sylvestris cv. Madre Negra,* to whom the inhibitory effect on the MCF7 cell line (breast cancer) was attributed.

The potential of this genus is not limited, since in recent years, the hybrid (H387) obtained from *Sechium edule* (*var. virens levis* × *var. amarus sylvestris*) had the presence of CuD, CuE, and CuB with predominating CuB, which may be involved in the strong antiproliferative and apoptotic potential in leukemic cell lines P388, J774, and WEHI-3 without affecting normal mouse bone marrow mononuclear cells (BM-MNC) [138].

Plants, as a rich source of cucurbitacins, amplify the potential of pharmacokinetic investigations since the descriptions of these characteristics are necessary to provide an optimal therapeutic design with the minimum toxicological damage to achieve adequate clinical management, and complete knowledge on its mechanism of action, side effects, and interactions with other drugs is crucial [18,139]. It has been described that some cucurbitacins produce transcendental changes related to the absorption of other drugs, as is the case with CuE, which increases the bioavailability of warfarin, triggering an increase in its coagulation potential, because CuE may inhibit metabolic processes by interrupting the activity of enzymes such as P450 or drug transporters such as P-glycoprotein, which indirectly affects the bioavailability of this drug [140]. This was also confirmed with the study carried out by Lu et al. (2017) [141], where CuE interrupted the activities of CYP3A and P-gp and led to a change in the pharmacokinetics of drugs such as indinavir to increase its C_max_ and AUC under an acute dose, unlike a successive treatment of three days of CuE that produced a decrease in the concentration of C_max_ and AUC of indinavir in in vivo models. Moreover, CuB, D, and E exhibited the ability to increase the interaction between albumin and ibuprofen, especially increasing the constant values of binding of warfarin to albumin without altering other binding sites of ligands [29].

Currently, pharmacokinetic studies in natural products have been a challenge, because this implies the presence of complex mixtures of substances that, in many cases, constitute unknown components [18], which is why these studies with phytopharmaceuticals remain limited [142]. Perhaps for this reason, it is easier to find research on pharmacokinetic profiles of commercially obtained compounds. Table 4 indicates the content of different types of cucurbitacins in different species of non-tropical genera, and Table 5 highlights the total content of cucurbitacins in different varietal groups of *Sechium edule*, with the yellow genotypes having concentrations up to 100 times lower than those of its wild relatives and 10 times smaller with respect to the green edible variety, which could be due to a change in the biosynthetic route of this compound in relation to the coevolution with humans [143].

## 6. Conclusions

Cucurbitacins are pharmacologically useful compounds, and their use as anticancer drugs offers great therapeutic expectations. This review integrates recent studies of their mechanisms of action and the pharmacokinetic profiles of the most studied cucurbitacins in vitro and in vivo. However, despite the availability of information regarding the molecular processes related to the biological action of these compound positions, it should be considered that there are still limitations regarding their pharmacokinetics. Determining the number of active ingredients in pharmacokinetic studies is essential, as this could help improve the design of strategies for ensuring their adequate therapeutic dosage as an antineoplastic agent. There is a wide range of plant species, especially neotropical species, from which metabolites originate and have not yet been explored.

## Figures and Tables

**Table 1:** Pharmacokinetic profiles of cucurbitacin IIa, IIb, B, and E.

Compound	Source	Model/Mode of Administration	Dose	Pharmacokinetic Parameters	Reference
CuIIa	*Hemsleya* *amabilis* extract	Normal rats/Oral administration	1.36 g/kg	C_max_ = 0.021 ± 0.0057 mg/L	[24]
				T_max_ = 0.333 ± 0.183 h	
				AUC_(0-t)_ = 21.36 ± 5.60 ng/h/mL	
				AUC_(0-__∞__)_ = 21.76 ± 5.60 ng/h/mL	
				MRT_(0–t)_ = 1.01 ± 0.23 h	
				MRT_(0–__∞__)_ = 1.08 ± 0.23 h	
				T_1/2_ = 0.678 ± 0.219 h	
				CL_z_ = 7209.68 ± 1805.38 L/h/kg	
CuIIa	Commercial sources	Rhesus monkeys /Intravenous injection	0.18 mg/kg	C_max_ = 1.3565 ± 0.12868 mg/L	[25]
				T_1/2Z_ = 0.455 ± 0.117 h	
				V_Z_ = 0.844 ± 0.299 L/kg	
				CL_z_ = 1.265 ± 0.149 L/h/kg	
				AUC_(0–__∞__)_ = 142.328 ± 16.392 μg/h/L	
				MRT = 0.338 ± 0.040 h	
CuIIa	Commercial sources	Wistar rats/Intravenous injection	1.0 mg/kg	C_max_ = 3.537 ± 0.278 mg/L T_1/2α_ = 0.073 ± 0.042 h T_1/2β_ = 0.732 ± 0.151 h T_1/2Z_ = 1.168 ± 0.415 h V_d_ = 0.147 ± 0.089 L/kg CL_z_ = 0.287 ± 0.031 L/h/kg AUC_(0–t)_ = 2.824 ± 0.578 mg/h/L AUC_(0–∞)_ = 3.646 ± 1.124 mg/h/L MRT = 0.479 ± 0.038 h	[28]
CuIIa	Commercial sources	Wistar rats /Intravenous injection	2.0 mg/kg	C_max_ = 6.452 ± 0.867 mg/L T_1/2α_ = 0.068 ± 0.031 h T_1/2β_ = 0.681 ± 0.055 h T_1/2Z_ = 0.985 ± 0.351 h V_d_ = 0.131 ± 0.095 L/kg CL_z_ = 0.304 ± 0.063 L/h/kg AUC_(0–t)_ = 4.133 ± 0.829 mg/h/L AUC_(0–∞)_ = 4.916 ± 1.227 mg/h/L MRT = 0.553 ± 0.054 h	[28]
CuIIa	Commercial sources	Wistar rats/Intravenous injection	4.0 mg/kg	C_max_ = 12.231 ± 2.77 mg/L T_1/2α_ = 0.074 ± 0.052 h T_1/2β_ = 0.667 ± 0.064 h T_1/2Z_ = 1.127 ± 0.614 h V_d_ = 0.153 ± 0.047 L/kg CL_z_ = 0.318 ± 0.029 L/h/kg AUC_(0–t)_ = 7.916 ± 0.582 mg/h/L AUC_(0–∞)_ = 9.385 ± 1.419 mg/h/L MRT = 0.517 ± 0.067 h	[28]
CuIIb	*Hemsleya* *amabilis* extract	Normal rats/Oral administration	1.36 g/kg	C_max_ = 0.02103 ± 0.00672 mg/L T_max_ = 0.667 ± 0.211 h AUC_(0–t)_ = 37.63 ± 13.01 ng/h/L AUC_(0–∞)_ = 38.54 ± 13.05 mg/h/L MRT_(0–t)_ = 1.35 ± 0.20 h MRT_(0–∞)_ = 1.48 ± 0.23 h T_1/2_ = 0.907 ± 0.349 h CL_z_ = 1553.35 ± 489.41 L/h/kg	[24]
CuB	*Trichosanthes* *Cucumerina* extract	Wistar rats/Oral administration	2.0 mg/kg	C_max_ = 0.0097 ± 0.0039 mg/L T_max_ = 0.50 ± 0.00 h AUC_0–t_ = 15.10 ± 3.57 μg/h/L AUC_0–inf_ = 25.33 ± 12.13 μg/h/L V_d_ = N/A L/kg CL = N/A L/h/kg T_1/2_ = N/A h MRT = 9.95 ± 12.27 h	[21]
CuB	*Trichosanthes* *cucumerina* extract	Wistar rats/Intravenous injection	0.1 mg/kg	C_max_ = N/A T_max_ = N/A AUC_(0–t)_ = 13.92 ± 11.11 μg/h/L AUC_0–inf_ = 17.95 ± 13.21 μg/h/L V_d_ = 51.65 ± 39.16 L/kg CL = 7.24 ± 2.92 L/h/kg T_1/2_ = 5.08 ± 2.87 h MRT = 6.03 ± 2.93 h	[21]
CuB	*Trichosanthes* *cucumerina* extract	Wistar rats/Oral administration	4.0 mg/kg	C_max_ = 0.03124 ± 0.0105 mg/L T_max_ = 0.60 ± 0.22 h AUC_(0–t)_ = 45.22 ± 10.14 μg/h/L AUC_0–inf_ = 52.42 ± 29.58 μg/h/L V_d_ = N/A L/kg CL = N/A L/h/kg T_1/2_ = N/A h MRT = 5.50 ± 2.28 h	[21]
CuB	Commercial sources	Wistar rats/Oral administration	20 mg/kg	C_max_ = 0.0059 ± 0.00101 mg/L T_max_ = 1.75 ± 0.88 h T_1/2_ = 2.50 ± 0.58 h AUC_last_ = 0.022 ± 0.005 mg/h/L AUC_inf_ = 0.024 ± 0.005 mg/h/L CL = 845.99 ± 183.70 L/h/kg	[20]
CuB	Commercial sources (Tablets)	Sprague Dawley rats/Oral administration	0.09 mg/kg	C_max_ = 0.030 ± 0.007 mg/L T_max_ = 2.41 ± 0.42 h K_e_ = 0.22 ± 0.04 T_1/2_ = 3.19 ± 0.54 h AUC_(0–t)_ = 140.4 = 2 ± 31.35 ng/h/L AUC_(0–∞)_ = 152.01 ± 35.02 ng/h/L	[26]
CuB	Commercial sources (Tablets)	Wistar rats/Oral administration	8 mg/kg	C_max_ = 3.41 × 10^−5^ ± 0.0029 mg/L T_max_ = 3 h T_1/2z_ = 4.129 ± 0.54 h AUC = _(0–t)_ = 183.28 ± 10.24 ng/L/h AUC_(0–∞)_ = 187.41 ± 10.41 ng/L/h *V*_z_/*F* = 2.55 × 10^8^ ± 3.62 × 10^7^ CL_z_/*F* = 4.28 × 10^7^ ± 2.47 × 10^6^ MRT_(0–t)_ = 6.49 ± 0.18 h MRT_(0–∞)_ = 7.02 ± 0.29 h	[27]
CuE	Commercial sources (Tablets)	Sprague Dawley rats/Oral administration	0.09 mg/kg	C_max_ = 0.009 ± 0.0026 mg/L T_max_ = 2.10 ± 0.21 h K_e_ = 0.23 ± 0.05 T_1/2_ = 2.58 ± 0.66 h AUC_(0–t)_ = 63.56 ± 11.92 ng/h/L AUC_(0–∞)_ = 67.27 ± 11.31 ng/h/L	[26]

Note: C_max_: maximum plasma concentration, T_1/2_: terminal elimination half-life, V_d_: apparent volume of distribution, T_max_: the time to reach C_max_, CL_z_/F: clearance = dose/AUC, AUC_(0–t)_ and AUC_(0–∞),_ area under the plasma concentration-time curve; MRT_(0–t)_ and MRT_(0–∞),_ mean residence time, k_e_ = elimination rate constant and V_z_ (apparent distribution volume = CLz/Zeta).

**Table 2:** Source and biological activity of cucurbitacin B.

Source	Biological Activity	Reference
*Ecballium elaterium*	Anti-inflammatory	[74]
*Citrullus colocynthis*	Cytotoxic in breast cancer	[15]
*Ecballium elaterium*	Anti-inflammatory	[75]
*Ecballium elaterium*	Antiproliferative	[76]
*Trichosanthes cucumerina*	Cytotoxic in breast cancer cells	[77]
*Cucumis prophetarum*	Cytotoxic in embryonal cancer	[78]
*Luffa operculata*	Antiproliferative, genotoxic activities in human breast cancer cells	[79]
*Leucopaxillus gentianeus*	Cytotoxic in breast cancer cells	[80]
*Cucurbita pepo*	Anti-inflammatory	[81]
*Trichosanthes cucumerina*	Cytotoxic in breast cancer	[82]
*Ecballium elaterium*	Hepatoprotective	[83]
*Begonia nantoensis*	Cytotoxic in multiple cancers	[84]
*Luffa graveolense*	Cytotoxic in lung cancer	[69]
*Cucumis callous*	Hypoglycemic	[85]
*Trichosanthes kirilowii*	Cytotoxic in liver cancer	[86]
*Momordica charantia*	Anti-inflammatory and antidiabetic	[87]
*Luffa operculata*	Antiproliferative in gastric adenocarcinoma cell line	[79]
*Aquilaria sinensis*	Cytotoxic	[88]

**Table 3:** Source and registry of the biological activity of cucurbitacin E.

Source	Biological Effect	Reference
*Ecballium elaterium*	Immunomodulatory	[102]
Commercial source	Cytotoxic enchondrosarcoma	[32]
*Citrullus lanatus*	Anti-inflammatory	[4]
*Ecballium elaterium*	Neuroprotective	[103]
*Ecballium elaterium*	Cytotoxic in human stomach adenocarcinoma cell line	[104]
*Ecballium elaterium*	Cytotoxic in ovarian cancer	[105]

**Table 4:** Content of cucurbitacins B, D, E, and I (mg/g) in different neotropical plant species.

Species	Parts of the Plant	CuB	CuD	CuE	CuI	Reference
*Cucurbita maxima*	Radicle	0.1–1	Trace	0.01–0.1	ND	[135,136]
	cotyledons	0.1–1	0.1–1	0.01–0.1	ND	
	leaf, fruit, root	>0.02	>0.02	>0.02	>0.02	
*Cucurbita andreana*	Sheet	0.15	0.12	ND	ND	[136]
	Fruit	2.78	2.78	ND	ND	
	Root	0.58	0.58	ND	ND	
*Cucurbita pepo*	Radicle	Trace	ND	0.1–1	Trace	[135]
	cotyledons	0.1–1	0.01–0.1	0.01–0.1	NI	
	Fruit	ND	ND	3.1	NI	
*Cucurbita martinezii*	Sheet	ND	ND	0.42	0.25	[136]
	Fruit	ND	ND	0.36	0.45	
	Root	ND	ND	0.23	0.65	
*Cucurbita lundelliana*	Sheet	0.47	0.12	ND	NI	[136]
	Fruit	0.63	0.15	ND	NI	
	Root	0.53	0.29	ND	NI	
*Cucurbita foetidissima*	Root	ND	ND	0.28	1.72	[136]
*Cucurbita equadorensis*	Placenta	0.14	0.5	0.03	0.06	[144]
	Pulp	0.006	0.01	Trace	Trace	
	Cortex	0.02	0.02	Trace	Trace	
*Sechium edule* var. *virens levis*	Fruit	ND	3.534	0.003	ND	[130]
*Sechium edule* var. *virens levis*	Fruit	0.0016	3.95	0.03	0.003	[130]
*Sechium edule* var. *nigrum spinosum*	Fruit	0.24	ND	0.05	ND	[130]
*Sechium edule* var. *nigrum spinosum*	Fruit	0.001	1.34	0.02	0.002	[130]
*Sechium* hybrid H387	Fruit	1.63	1.62	0.42	0.088	[145]

Note: ND = Not detected.

**Table 5:** Total content of cucurbitacins (mg/g) in different genotypes of *Sechium edule* species.

Species	Parts of the Plant	Total Content of Cucurbitacins	Reference
*Sechium edule* var. *nigrum xalapensis*	Fruit	0.00195	[146]
*Sechium edule* var. *nigrum levis*	Fruit	0.00066	[146]
*Sechium edule* var*. amarus sylvestris*	Fruit	0.001456	[146]
*Sechium edule* var. *albus minor*	Fruit	3.9 × 10^−5^	[146]
*Sechium edule* var. *albus dulcis*	Fruit	2.7 × 10^−5^	[146]
*Sechium edule* var. *albus levis*	Fruit	8.8 × 10^−5^	[146]

## Data Availability

Data sharing not applicable.
